# Impact of cardiac rehabilitation on ventricular-arterial coupling and left ventricular function in patients with acute myocardial infarction

**DOI:** 10.1371/journal.pone.0300578

**Published:** 2024-04-04

**Authors:** Ho-Min Yoon, Seung-Jae Joo, Ki Young Boo, Jae-Geun Lee, Joon-Hyouk Choi, Song-Yi Kim, So Young Lee

**Affiliations:** 1 Department of Rehabilitation Medicine, Jeju National University Hospital, Jeju, Republic of Korea; 2 Department of Internal Medicine, Jeju National University Hospital, Jeju, Republic of Korea; 3 Department of Internal Medicine, Jeju National University College of Medicine, Jeju, Republic of Korea; 4 Department of Rehabilitation Medicine, Jeju National University College of Medicine, Jeju, Republic of Korea; University of Southern California, UNITED STATES

## Abstract

To maintain efficient myocardial function, optimal coordination between ventricular contraction and the arterial system is required. Exercise-based cardiac rehabilitation (CR) has been demonstrated to improve left ventricular (LV) function. This study aimed to investigate the impact of CR on ventricular-arterial coupling (VAC) and its components, as well as their associations with changes in LV function in patients with acute myocardial infarction (AMI) and preserved or mildly reduced ejection fraction (EF). Effective arterial elastance (E_A_) and index (E_A_I) were calculated from the stroke volume and brachial systolic blood pressure. Effective LV end-systolic elastance (E_LV_) and index (E_LV_I) were obtained using the single-beat method. The characteristic impedance (Zc) of the aortic root was calculated after Fourier transformation of both aortic pressure and flow waveforms. Pulse wave separation analysis was performed to obtain the reflection magnitude (RM). An exercise-based, outpatient cardiac rehabilitation (CR) program was administered for up to 6 months. Twenty-nine patients were studied. However, eight patients declined to participate in the CR program and were subsequently classified as the non-CR group. At baseline, E’ velocity showed significant associations with E_A_I (beta -0.393; *P* = 0.027) and VAC (beta -0.375; *P* = 0.037). There were also significant associations of LV global longitudinal strain (LV GLS) with E_A_I (beta 0.467; *P* = 0.011). Follow-up studies after a minimum of 6 months demonstrated a significant increase in E’ velocity (*P* = 0.035), improved EF (*P* = 0.010), and LV GLS (*P* = 0.001), and a decreased E_A_I (*P* = 0.025) only in the CR group. Changes in E’ velocity were significantly associated with changes in E_A_I (beta -0.424; *P* = 0.033). Increased aortic afterload and VA mismatch were associated with a negative impact on both LV diastolic and systolic function. The outpatient CR program effectively decreased aortic afterload and improved LV diastolic and systolic dysfunction in patients with AMI and preserved or mildly reduced EF.

## Introduction

Acute myocardial infarction (AMI) is the leading cause of cardiac death and heart failure (HF) worldwide [1,2]. After receiving initial emergency care and reperfusion therapy, evidence-based long-term treatment is necessary to improve clinical outcomes. To prevent the progression of HF, hospitalization due to HF and cardiac death in patients with AMI, comprehensive cardiac rehabilitation (CR) is strongly recommended in the guidelines [3,4]. This rehabilitation program includes exercise training, the management and control of cardiovascular risk factors, and dietary advice [5].

Patients with AMI often demonstrate increased arterial stiffness, which can have adverse effects on both LV systolic and diastolic function. As arterial stiffness increases, both forward and reflected wave velocities accelerate. This causes the reflection wave to overlap in mid-systole, resulting in elevated aortic systolic and pulse pressure, accompanied by a decrease in diastolic pressure. The amplified aortic systolic pressure raises LV afterload and myocardial oxygen demand. Simultaneously, the diminished aortic diastolic pressure reduces coronary artery blood flow, potentially inducing myocardial ischemia. These effects are more pronounced when LV systolic function is preserved [6–9]. In this context, it’s essential to ensure optimal coordination between ventricular contraction and the arterial system through which blood is pumped to maintain efficient myocardial function. Abnormal coupling between these factors is implicated in the pathogenesis of HF [8,9].

Arterial load was characterized in the frequency domain, while left ventricular (LV) systolic function was evaluated using pressure-volume (PV) loop indices in the time domain. However, assessing the direct interaction between LV contractile function and arterial load was challenging because they were typically expressed in different units. Therefore, elastance, which measures the increase in pressure with volume change, is commonly used to estimate ventricular-arterial coupling (VAC) despite its inherent limitations [10,11]. The ratio of effective arterial elastance (E_A_) to effective LV end-systolic elastance (E_LV_) is usually used as the measure of VAC.

Exercise-based cardiac rehabilitation (CR) has been demonstrated to enhance exercise capacity, as measured by peak oxygen uptake, in patients with HF or AMI [12,13]. Additionally, in patients with heart failure and reduced ejection fraction (EF), an increase in peak oxygen uptake has been associated with improved clinical outcomes [12]. The functional improvement of myocardial contractility after CR in patients with AMI is typically assessed by evaluating changes in LV volume and EF using echocardiography [13]. While the measurement of VAC and its component can provide incremental insight into LV functional change after CR in patients with AMI and preserved or mildly reduced EF, it has been the subject of limited investigation. This study aimed to investigate the impact of CR on VAC and LV function in patients with AMI.

## Methods

### Study patients

Patients who were hospitalized for AMI, underwent successful coronary reperfusion, were scheduled a comprehensive CR program, and agreed to take part in the study were consecutively enrolled from June 25, 2020 to May 31, 2021. Exclusion criteria included patients with reduced EF ≤40%, valvular heart diseases, thyroid diseases, a history of stroke within one year, or those not in sinus rhythm. Patients who did not have follow-up echocardiographic or hemodynamic data were also excluded from the study. This study was conducted in accordance with the Declaration of Helsinki. The study protocol received approval from the institutional review board (IRB) at Jeju National University Hospital, Republic of Korea (IRB No. JNUH-2020-02-007). Written informed consents were obtained from participating patients. Patients who did not participate in the outpatient CR program were categorized as the non-CR group. Age, gender, height, weight, body surface area (BSA), estimated glomerular filtration rate (eGFR) and co-morbidities such as hypertension, diabetes mellitus, or angina, as well as past medical history of myocardial infarction (MI), HF, or stroke, smoking status, type of AMI, modality of coronary reperfusion, culprit lesions identified in coronary angiography, and medications at discharge, were all collected from electronic medical records.

### Transthoracic echocardiographic study

Transthoracic echocardiographic studies were conducted using the Vivid E95 system (GE Medical, Milwaukee, WI, USA). LV wall thickness (cm), LV end-diastolic and end-systolic dimensions (cm), and left atrium (LA) dimension (cm) were measured from the M-mode tracings. LA end-systolic volume (mL) was determined using the biplane method of discs. LV mass index (gram/m^2^) was calculated using the Devereux formula [14]. LV volumes (mL) in diastole and systole were assessed from apical 4- and 2-chamber views using modified Simpson’s method, and LV EF (%) was computed.

Standard diastolic filling parameters such as peak early-diastolic (E wave) and peak late-diastolic (A wave) transmitral flow velocities (cm/sec), E/A ratio, early-diastolic (E’ wave), late-diastolic (A’ wave), and systolic (S’ wave) septal mitral annular velocities (cm/sec) along with E/E’ ratio were measured at the apical 4-chamber view using pulsed and tissue Doppler echocardiographic images. Stroke volume (SV, in mL) was calculated as the product of LV outflow tract area (cm^2^) measured at the parasternal long-axis and the time-velocity integral (cm) of LV outflow tract (LVOT) flow acquired by pulsed-wave Doppler echocardiography at the apical 5-chamber view. Cardiac output (CO, in L/min) was determined as SV (mL) × heart rate (HR, in beats/min) / 1000, and indexed by BSA (CI, in L/min/m^2^). Right ventricular (RV) systolic pressure (mmHg) was estimated from the peak systolic velocity (m/sec) of tricuspid regurgitant flow in the continuous-wave Doppler image at RV inflow view.

LV strain analysis was conducted using 2-dimensional speckle tracking images in the apical 2-, 3-, and 4-chamber views with vendor-provided software. The mean LV global longitudinal strain (GLS, in %) was subsequently calculated.

### Hemodynamic study

Hemodynamic data was acquired in the supine position following a transthoracic echocardiographic examination. Since hemodynamic parameters are influenced by body size, they were indexed by BSA [11]. Brachial blood pressure (BP, in mmHg) measurements were obtained using digital sphygmomanometer (Microlife BP A100, Microlife AG, Widnau, Switzerland). Brachial pulse pressure (PP, in mmHg) was calculated as the difference between brachial systolic BP (SBP) and brachial diastolic BP (DBP). Mean brachial BP (mmHg) was determined as brachial PP/3 + brachial DBP. Systemic vascular resistance (SVR, in dynes/sec·cm^-5^) was computed as mean brachial BP multiplied by 80, divided by CO, and indexed by BSA (SVRI, in dynes/sec·cm^-7^). Central aortic pressures were estimated through pressure wave analysis (PWA) of pressure waveform at the radial artery using the applanation tonometry (SphygmoCor®, AtCor, Sydney, Australia). The radial pressure waveform was calibrated using brachial SBP and DBP. From this PWA, central (proximal aortic) SBP (mmHg), central DBP, central PP (mmHg), central end-systolic pressure (ESP, in mmHg), aortic augmentation index (AIx, in %), AIx adjusted to a heart rate 75 beats per minute (AIx75, in %), and pressure-time indexes (mmHg·sec/min) at systole (sPTI) and at diastole (sPTI) were measured. Total arterial compliance (TAC, in mL/mmHg) was calculated as the following equation and indexed by BSA (TACI, in mL/mmHg·m^2^): TAC = (dPTI × SV) / [(sPTI + dPTI) × (central ESP—central DBP)] [15,16].

### Measurements of VAC and its components

ESP was determined as 0.9 times brachial SBP [17]. E_A_ (mmHg/mL) was calculated as ESP divided by SV and indexed by BSA (E_A_I in mmHg/mL·m^2^). E_LV_ (mmHg/mL) was estimated using the single-beat method, approximating it from time-varying elastance curve [18–20]. E_LV_ was calculated using the following formula:

E_LV_ = [Brachial DBP—(E_Nd(est)_ × ESP)] / (SV × E_Nd(est)_)

Here, E_Nd(est)_ represents the normalized LV elastance at the onset of ejection and is determined by the formula:

E_Nd(est)_ = 0.0275–0.165 × EF + 0.3656 × (Brachial DBP / ESP) + 0.515 × E_Nd(avg)_

E_Nd(avg)_ is calculated as:

E_Nd(avg)_ = 0.35695–7.2266 × tNd + 74.249 × tNd^2^–307.39 × tNd^3^ + 684.54 × tNd^4^–856.92 × tNd^5^ + 571.95 × tNd^6^–159.1 × tNd^7^

Here, tNd represents the ratio of the pre-ejection period to the total systolic period. tNd was acquired from the pulsed-wave Doppler tracing of LVOT flow at the apical 5-chamber view as the ratio of the period from ECG Q wave to flow-onset to the period from ECG Q wave to end-flow (S1 Fig). E_LV_ (mmHg/mL) was indexed by BSA (E_LV_I in mmHg/mL·m^2^). VAC was determined as the ratio of E_A_ to E_LV_.

### Measurements of aortic characteristic impedance and reflection magnitude

Digitized data of aortic pressure, estimated from the radial waveform, and digitized data of LVOT flow, acquired from pulsed-wave Doppler echocardiography at the apical 5-chamber view, were used for aortic pressure-flow analysis. The software for aortic pressure-flow analysis was self-programmed using LabVIEW (National Instruments, Austin, TX, USA).

Systolic ejection period was synchronized by aligning the rapid increase in aortic pressure wave with beginning of LVOT flow and the dicrotic notch of aortic pressure with the cessation of LVOT flow. Aortic input impedance (Zin) was calculated as the ratio of the modulus of aortic pressure to LVOT flow in the frequency domain after Fourier transformation. Aortic characteristic impedance (Zc) was determined as the average value of the 3rd to 10th harmonics of Zin. Next, wave separation analysis was performed using Zc to obtain reflection magnitude (RM). The forward pressure wave (Pf) and backward pressure wave (Pb) was calculated as follows (S2 Fig):

Pf = (P + Q × Zc) / 2, and Pb = (P − Q × Zc) / 2, where P represents pressure and Q represents flow.

RM was defined as Pb/Pf.

### Comprehensive cardiac rehabilitation program

During the inpatient stay, patients received education about the general CR program and learned about controlling risk factors to prevent recurrent MI. Patients who agreed to participate in the outpatient CR program initially underwent a medical checkup by cardiologists two weeks after discharge. Their cardiorespiratory fitness was evaluated using a treadmill exercise stress test. Based on these results, they were categorized into low-, moderate-, or high-risk group and then prescribed exercise type, frequency, intensity, and duration accordingly. The outpatient CR program included both aerobic and resistance exercises. The aerobic exercise segment comprised warm-up, treadmill activities, and cool-down exercises. Resistance exercises focused on leg workouts such as squats, half squats, lunges, and calf raises. Exercise intensity was set to fall between 11 and 13 on the rating of perceived exertion scale. Exercise intensity and duration were gradually increased, considering both the exercise prescription and the patient’s response. A total of 18 sessions were conducted over a period of 4 to 6 weeks. Home-based CR was prescribed to patients who completed the outpatient CR program. It was also offered to patients who preferred it over hospital-based CR.

### Follow-up studies

Follow-up echocardiographic and hemodynamic studies were conducted after a minimum of 6 months, employing the same protocols as those used at baseline.

### Statistical analysis

Data were presented as median (interquartile range) for continuous variables, and as a number (percentage) for categorical variables. Median values between the CR group and the non-CR group were compared using the Mann-Whitney U test, while categorical variables were compared using the Chi-square test. Changes in echocardiographic and hemodynamic parameters during the follow-up studies were analyzed using the Wilcoxon signed rank test. The associations of E’ velocity and LV GLS with hemodynamic data were assessed through correlation and linear regression analysis. Additionally, the association of changes in E’ (ΔE’) and LV GLS (ΔLV GLS) with changes in VAC and its components were evaluated using correlation and linear regression analysis. All statistical analyses were conducted using the statistical package SPSS version 23 (IBM Co, Armonk, NY, US). Clinical significance was defined as a *P*-value <0.05.

## Results

A total of 90 patients with AMI were consecutively enrolled. After excluding 5 patients with a significant aortic stenosis and 56 patients lacking follow-up echocardiographic or hemodynamic data, 29 patients were included in this study. Among them, 8 patients did not participate in the outpatient CR program. They were classified as the non-CR group. Other 21 patients constituted the CR group.

### Baseline clinical characteristics

Age, gender, height, BMI, BSA, co-morbidities such as hypertension, diabetes mellitus, or angina, past-medical history of MI, HF, or stroke, current smoking status, and eGFR were not different between the two groups. The percentage of ST-elevation MI and non-ST-elevation MI was similar between the two groups. All patients underwent percutaneous coronary intervention as the modality of coronary reperfusion, with coronary stents implanted in all cases except for one patient in the non-CR group. Antiplatelet agents, beta-blockers, renin-angiotensin system inhibitors, calcium channel blocker, nitrate and statins were prescribed similarly at discharge (Table 1).

**Table 1:** Baseline characteristics of patients.

	Total (N = 29)	With CR (N = 21)	Without CR (N = 8)	*P* value
Age (years)	60 (51, 66)	59 (51, 66)	62 (51, 70)	0.549
Male	25 (86.2)	18 (85.7)	7 (87.5)	1.000
Height (cm)	168 (164, 174)	168 (164, 175)	171 (163, 173)	1.000
Weight (kg)	75 (68, 80)	76 (71, 82)	72 (64, 77)	0.237
Body mass index (kg/m^2^)	26.4 (24.1, 28.4)	26.9 (24.3, 28.5)	25.0 (23.9, 26.6)	0.200
Body surface area (/m^2^)	1.86 (1.75, 1.95)	1.86 (1.78, 1.97)	1.81 (1.73, 1.90)	0.349
Hypertension	12 (41.4)	7 (33.3)	5 (62.5)	0.218
Diabetes mellitus	10 (34.5)	7 (33.3)	3 (37.5)	1.000
Angina	3 (10.3)	1 (4.8)	2 (25.0)	0.176
Prior myocardial infarction	1 (3.4)	1 (4.8)	0 (0)	1.000
Prior heart failure	1 (3.4)	1. (4.8)	0 (0)	1.000
Stroke	3 (10.3)	2 (9.5)	1 (12.5)	1.000
Smoker	13 (44.8)	8 (38.1)	5 (62.5)	0.406
eGFR	91.8 (80.7, 103.9)	91.8 (80.2, 103.1)	93.3 (81.2, 106.3)	0.684
eGFR <60 mL/min/1.73m^2^	2 (6.9)	1 (4.8)	1 (12.5)	0.483
STEMI	18 (62.1)	14 (66.7)	4 (50.0)	0.433
PCI	29 (100)	21 (100)	8 (100)	
PCI with stents	28 (96.6)	21 (100)	7 (87.5)	0.276
LAD	13 (44.8)	9 (42.9)	4 (50.0)	0.942
LCX	4 (13.8)	3 (14.3)	1 (12.5)	
RCA	12 (41,4)	9 (42.9)	3 (37.5)	
Medications				
Aspirin	28 (96.6)	20 (95.2)	8 (100)	1.000
P2Y12 inhibitors	29 (100)	21 (100)	8 (100)	
Beta-blockers	23 (79.3)	18 (85.7)	6 (62.5)	0.305
ACEI	1 (3.4)	0 (0)	1 (12.5)	0.276
ARB	13 (44.8)	7 (33.3)	6 (75.0)	0.092
Calcium channel blockers	3 (10.3)	2 (9.5)	1 (12.5)	1.000
Nitrate	4 (13.8)	3 (14.3)	1 (12.5)	1.000
Statins	29 (100)	21 (100)	8 (100)	

Values are median (interquartile range) or number (%).

ACEI, angiotensin converting enzyme inhibitor; ARB, angiotensin receptor blocker; CR, cardiac rehabilitation; eGFR, estimated glomerular filtration rate; LAD, left anterior descending artery; LCX, left circumflex artery; PCI, percutaneous coronary intervention; RCA, Right coronary artery; STEMI, ST-elevation myocardial infarction.

### Baseline echocardiographic and hemodynamic data

LV wall thickness, LV dimensions, relative wall thickness and LA end-systolic volume were not different between the two groups, while LV mass index was greater in the non-CR group. End-diastolic volume (EDV), end-systolic volume (ESV), SV, HR, CO, and CI were not different between the two groups. Median EF and LV GLS were 53.4% and -14.4%, respectively, and these values were not significantly different between the two groups. Approximately 70% of patients had an EF ≥50%, and this percentage was consistent across both groups. LV diastolic parameters including E velocity, A velocity, E/A ratio, E’ velocity, A’ velocity, S’ velocity and E/E’ ratio showed no significant difference between the two groups. RV systolic pressure also showed no difference (Table 2).

**Table 2:** Baseline echocardiographic data.

	Total (N = 29)	With CR (N = 21)	Without CR (N = 8)	*P* value
IVS (cm)	1.02 (0.98, 1.11)	1.01 (0.96, 1.04)	1.11 (1.00, 1.27)	0.059
LVPW (cm)	0.93 (0.86, 1.00)	0.93 (0.86, 0.99)	0.97 (0.86, 1.00)	0.401
LVDD (cm)	4.90 (4.68, 5.12)	4.84 (4.57, 5.06)	5.07 (4.92, 5.19)	0.083
LVSD (cm)	3.28 (2.98, 3.42)	3.25 (2.88, 3.43)	3.34 (3.24, 3.39)	0.582
LVMI (g/m^2^)	89.3 (82.7, 102.1)	86.7 (80.1, 97.1)	107.3 (87.8, 119.7)	0.041
RWT	0.38 (0.37, 0.40)	0.39 (0.36, 0.40)	0.38 (0.37, 0.40)	0.582
LAESVI (mL/m^2^)	35.2 (31.7, 42.2)	35.0 (31.1, 42.1)	40.3 (33.4, 56.8)	0.257
EDV (mL)	109.7 (85.3, 135.7)	109.7 (85.3, 134.0)	112.8 (87.8, 149.5)	0.684
ESV (mL)	48.7 (40.4, 59.0)	48.7 (40.3, 56.0)	52.3 (40.6, 67.9)	0.684
EF (%)	53.4 (49.3, 59.9)	52.9 (49.1, 59.9)	55.2 (52.6, 59.5)	0.720
EF ≥50%	20 (69.0)	13 (61.9)	7 (87.5)	0.371
LVGLS (%)	-14.4 (-15.9, -12.2)	-13.9 (-15.9, -11.7)	-14.8 (-15.7, -13.7)	0.374
LVOTd (cm)	2.17 (2.06, 2.30)	2.13 (2.04, 2.22)	2.27 (2.12, 2.33)	0.184
SV (mL)	65.3 (55.0, 74.1)	62.6 (53.3, 70.1)	71.7 (58.8, 87.3)	0.103
Heart rate (/min)	66 (57, 77)	69 (58, 80)	64 (56, 71)	0.184
CO (L/min)	4.25 (3.66, 4.97)	4.16 (3.44, 4.78)	4.83 (4.12, 5.00)	0.301
CI (L/min/m^2^)	2.28 (1.96, 2.85)	2.19 (1.90, 2.57)	2.80 (2.25, 2.90)	0.200
E velocity (cm/sec)	53.6 (47.9, 66.2)	54.2 (44.3, 69.1)	52.5 (50.2, 63.4)	0.582
A velocity (cm/sec)	72.9 (56.7, 84.2)	66.0 (56.0, 83.0)	78.7 (63.3, 95.3)	0.200
E/A ratio	0.69 (0.51, 1.06)	0.71 (0.51, 1.17)	0.68 (0.54, 0.97)	0.615
E’ velocity (cm/sec)	5.79 (5.05, 6.64)	5.95 (5.05, 6.75)	5.48 (3.85, 6.01)	0.429
A’ velocity (cm/sec)	8.64 (7.66, 10.30)	8.59 (7.28, 10.92)	9.03 (8.33, 10.09)	0.582
S’ velocity (cm/sec)	7.37 (5.98, 8.38)	7.67 (5.94, 8.66)	6.35 (5.99, 8.07)	0.457
E/E’	9.52 (6.67, 12.08)	9.26 (6.62, 11.50)	11.11 (7.25, 14.64)	0.200
RVSP (mmHg)	24.0 (21.1, 29.2)	24.1 (21.7, 29.2)	23,7 (19.2, 28.6)	0.549

Values are median (interquartile range) or number (%).

CI, cardiac index; CO, cardiac output; EDV, end-diastolic volume; EF, ejection fraction; ESV, end-systolic volume; IVS, interventricular septal thickness; LAESVI, left atrial end-systolic volume index; LVDD, left ventricular end-diastolic dimension; LVGLS, left ventricular global longitudinal strain; LVMI, left ventricular mass index; LVOTd, left ventricular outflow tract diameter; LVPW, left ventricular posterior wall thickness; LVSD, left ventricular end-systolic dimension; RVSP, right ventricular systolic pressure; RWT, relative wall thickness; SV, stroke volume

Brachial SBP and DBP, brachial PP, SVR, and SVRI were not significantly different between the two groups. Similarly, central SBP and DBP, central PP, TAC, TACI, AIx and AIx75 showed no significant difference. Median VAC was 1.0, and this value did not significantly differ between the two groups. E_LV_, E_LV_I, E_A_, and E_A_I were not significantly different between the two groups. Zc and RM also showed no significant difference between the two groups (Table 3).

**Table 3:** Baseline hemodynamic data.

	Total (N = 29)	With CR (N = 21)	Without CR (N = 8)	*P* value
Brachial SBP (mmHg)	119 (104, 128)	116 (104, 128)	123 (118, 127)	0.184
Brachial DBP (mmHg)	70 (66, 82)	70 (66, 81)	73 (65, 83)	0.867
PP (mmHg)	41 (37, 51)	40 (35, 49)	44 (41, 60)	0.168
SVR (dynes/sec/cm^-5^)	1646 (1403, 1866)	1730 (1403, 1911)	1552 (1403, 1726)	0.457
SVRI (dynes/sec/cm^-7^)	913 (773, 1006)	922 (777, 1006)	862 (742, 1068)	0.756
TAC (ml/mmHg)	1.82 (1.38, 2.07)	1.80 (1.43, 2.13)	1.83 (1.06, 2.04)	0.487
TACI (ml/mmHg∙m^2^)	0.96 (0.73, 1.15)	0.96 (0.77, 1.15)	0.99 (0.62, 1.17)	0.793
Central SBP (mmHg)	110 (96, 117)	108 (94, 115)	114 (108, 119)	0.083
Central DBP (mmHg)	71 (66, 83)	71 (67, 83)	74 (66, 83)	0.943
Central PP (mmHg)	33 (26, 42)	29 (24, 42)	36 (32, 49)	0.083
Heart rate (/min)	65 (58, 78)	65 (58, 78)	63 (55, 65)	0.257
AIx75 (%)	17.8 (13.4, 26.1)	17.8 (10.5, 25.1)	17.4 (13.4, 35.4)	0.615
E_LV_ (mmHg/ml)	1.61 (1.42, 1.85)	1.62 (1.46, 1.92)	1.61 (1.37, 1.71)	0.549
E_LV_I (mmHg/ml∙m^2^)	0.88 (0.78, 1.00)	0.88 (0.76, 1.04)	0.89 (0.79, 0.94)	1.000
E_A_ (mmHg/ml)	1.67 (1.34, 2.06)	1.69 (1.41, 2.07)	1.54 (1.27, 2.00)	0.429
E_A_I (mmHg/ml∙m^2^)	0.90 (0.75, 1.13)	0.90 (0.76, 1.13)	0.84 (0.68, 1.17)	0.649
VAC	1.00 (0.87, 1.20)	1.05 (0.87, 1.23)	0.95 (0.84, 1.17)	0.615
Zc (x 10^3^ dyne-sec/cm^3^)	0.180 (0.130, 0.289)	0.163 (0.125, 0.262)	0.278 (0.150, 0.401)	0.114
RM	0.83 (0.77, 0.86)	0.83 (0.78, 0.86)	0.82 (0.75, 0.88)	0.830

Values are median (interquartile range).

AIx75, augmentation index corrected at heart rate 75/min; DBP, diastolic blood pressure; E_A_, effective arterial elastance; E_A_I, effective arterial elastance index; E_LV_, left ventricular end-systolic elastance; E_LV_I, left ventricular end-systolic elastance index; PP, pulse pressure; RM, reflection magnitude; SBP, systolic blood pressure; SVR, systemic vascular resistance; SVRI, systemic vascular resistance index; TAC, total arterial compliance; TACI, total arterial compliance index; VAC, ventricular arterial coupling; Zc, characteristic impedance

### Associations of E’ velocity and LV GLS with hemodynamic data

**Fig 1:**
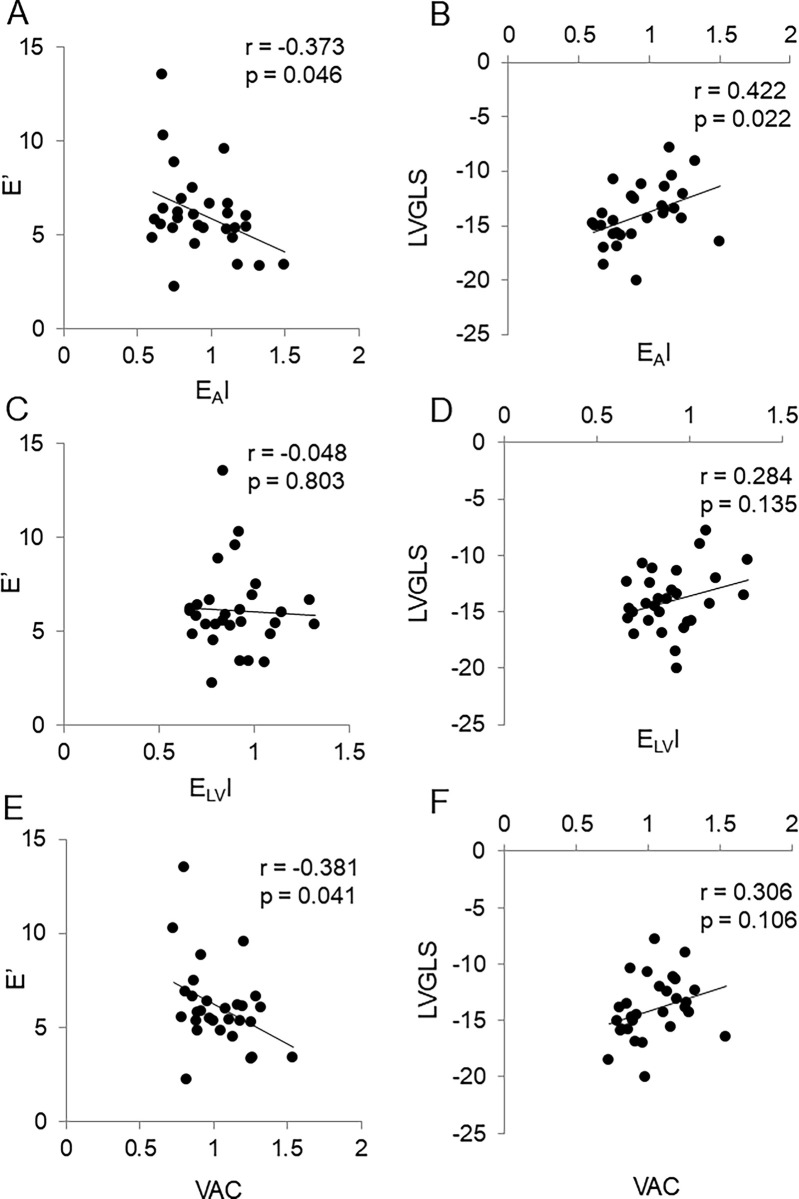
Correlations of E’ velocity (cm/sec) and left ventricular global longitudinal strain (LV GLS, in %) with hemodynamic data in the entire patient cohort (N = 29). (A) E’ velocity and effective arterial elastance index (E_A_I, in mmHg/ml∙m^2^). (B) LV GLS and E_A_I. (C) E’ velocity and effective left ventricular end-systolic elastance index (E_LV_I, in mmHg/ml∙m^2^). (D) LV GLS and E_LV_I. (E) E’ velocity and ventricular-arterial coupling (VAC). (F) LV GLS and VAC.

At baseline, E’ velocity showed a negative correlation with SVRI, E_A_I, and VAC, but not with E_LV_I (Fig 1). In linear regression analysis, adjusted for age and sex, E’ velocity exhibited significant associations with SVRI (beta -0.424; P = 0.014), E_A_I (beta -0.393; *P* = 0.027), and VAC (beta -0.375; *P* = 0.037) (Table 4).

**Table 4:** Linear regression analysis of baseline E’ and LVGLS with hemodynamic data (N = 29).

	E’				LVGLS			
	Unadjusted		Adjusted*		Unadjusted		Adjusted*	
	Beta	*P* value	Beta	*P* value	Beta	*P* value	Beta	*P* value
SVR	-0.209	0.277	-0.401	0.033	0.329	0.081	0.248	0.225
SVRI	-0.386	0.038	-0.424	0.014	0.332	0.078	0.311	0.097
TAC	0.164	0.396	0.128	0.496	-0.171	0.376	-0.244	0.210
TACI	0.080	0.679	0.094	0.609	-0.190	0.323	-0.200	0.294
E_LV_	0.134	0.488	-0.044	0.828	0.286	0.133	0.193	0.358
E_LV_I	-0.048	0.803	-0.100	0.587	0.284	0.135	0.265	0.163
E_A_	-0.260	0.173	-0.390	0.031	0.476	0.009	0.445	0.018
E_A_I	-0.373	0.046	-0.393	0.027	0.422	0.022	0.467	0.011
VAC	-0.381	0.041	-0.375	0.037	0.306	0.106	0.370	0.051
Zc	-0.157	0.416	-0.097	0.601	-0.090	0.643	-0.053	0.784
RM	-0.163	0.400	-0.061	0.751	0.164	0.395	0.233	0.238

*adjusted for age and sex

E_A_, effective arterial elastance; E_A_I, effective arterial elastance index; E_LV_, left ventricular end-systolic elastance; E_LV_I, left ventricular end-systolic elastance index; LVGLS, left ventricular global longitudinal strain; PWV, pulse wave velocity; SVR, systemic vascular resistance; SVRI, systemic vascular resistance index; TAC, total arterial compliance; TACI, total arterial compliance index; VAC, ventricular arterial coupling; Zc, characteristic impedance

Conversely, baseline LV GLS displayed positive correlations with E_A_ and E_A_I, but exhibited no correlation with E_LV_I or VAC (Fig 1). In linear regression analysis, also adjusted for age and sex, significant associations of LV GLS with E_A_ (beta 0.445; *P* = 0.018) and E_A_I (beta 0.467; *P* = 0.011) were demonstrated (Table 4).

### Follow-up echocardiographic and hemodynamic data

Follow-up echocardiographic and hemodynamic studies were conducted at a median 272 days after the initial assessments, and this duration did not significantly differ between the two groups. LV wall thickness, LV dimensions, LV mass index, relative wall thickness, LA end-systolic volume, EDV, ESV, SV, HR, CO, and CI were not significantly different between the two groups. Median EF and LV GLS were 57.0% and -15.7%, respectively, and these values did not significantly differ between the two groups. LV diastolic parameters including E velocity, A velocity, E/A ratio, E’ velocity, A’ velocity, S’ velocity, and E/E’ ratio also showed no significant differences between the two groups. RV systolic pressure exhibited no difference (S1 Table).

When compared with the baseline echocardiographic data, SV, EF, E/A ratio, and E’ velocity were significantly increased, while ESV, LV GLS, and HR were decreased in the entire patient cohort (S2 Table). However, improvements in E’ velocity, EF, and LV GLS were found to be statistically significant only in the CR group (median E’ velocity from 5.95 cm/sec to 6.60 cm/sec; *P* = 0.035, median EF from 52.9% to 58.3%; *P* = 0.010, and median LV GLS from -13.9% to -15.9%; *P* = 0.001). Additionally, the change in LV GLS was greater in the CR group (median value -2.6% vs. -0.1%; *P* = 0.011) (Table 5).

**Table 5:** Changes of echocardiographic data.

	With CR (N = 21)		Without CR (N = 8)	
	Baseline	Follow up	Baseline	Follow up
IVS (cm)	1.01 (0.96, 1.04)	0.98 (0.89, 1.05)	1.11 (1.00, 1.27)	1.03 (0.99, 1.31)
LVPW (cm)	0.93 (0.86, 0.99)	0.86 (0.82, 0.95)	0.97 (0.86, 1.00)	0.97 (0.88, 1.00)
LVDD (cm)	4.84 (4.57, 5.06)	4.87 (4.52, 5.23)	5.07 (4.92, 5.19)	5.05 (4.55, 5.17)
LVSD (cm)	3.25 (2.88, 3.43)	3.24 (2.90, 3.54)	3.34 (3.24, 3.39)	3.33 (2.93, 3.39)
LVMI (g/m^2^)	86.7 (80.1, 97.1)	88.4 (76.5, 97.6)	107.3 (87.8, 119.7)	105.2 (83.8, 124.4)
RWT	0.39 (0.36, 0.40)	0.36 (0.34, 0.39)	0.38 (0.37, 0.40)	0.39 (0.35, 0.41)
LAESVI (mL/m^2^)	35.0 (31.1, 42.1)	38.2 (32.0, 43.0)	40.3 (33.4, 56.8)	44.1 (37.5, 49.7)
EDV (mL)	109.7 (85.3, 134.0)	111.9 (99.8, 128.5)	112.8 (87.8, 149.5)	101.5 (81.1, 119.0)*
ESV (mL)	48.7 (40.3, 56.0)	47.8 (40.3, 58.4)	52.3 (40.6, 67.9)	42.4 (29.6, 58.8)
EF (%)	52.9 (49.1, 59.9)	58.3 (52.0, 62.2)*	55.2 (52.6, 59.5)	55.5 (52.3, 62.3)
LV GLS (%)	-13.9 (-15.9, -11.7)	-15.9 (-18.5, -14.8)*	-14.8 (-15.7, -13.7)	-14.0 (-16.7, -12.3)
SV (mL)	62.6 (53.3, 70.1)	75.9 (65.7, 83.4)*	71.7 (58.8, 87.3)	72.7 (70.8, 80.3)
Heart rate (/min)	69 (58, 80)	61 (55, 66)*	64 (56, 71)	60 (55, 67)
CO (L/min)	4.16 (3.44, 4.78)	4.56 (3.66, 5.20)	4.83 (4.12, 5.00)	4.62 (4.06, 4.99)
CI (L/min/m^2^)	2.19 (1.90, 2.57)	2.43 (2.01, 2.86)	2.80 (2.25, 2.90)	2.44 (2.24, 3.12)
E velocity (cm/sec)	54.2 (44.3, 69.1)	63.2 (53.4, 72.9)*	52.5 (50.2, 63.4)	45.7 (36.8, 72.3)
A velocity (cm/sec)	66.0 (56.0, 83.0)	71.7 (60.0, 82.8)	78.7 (63.3, 95.3)	68.8 (59.1, 82.0)
E/A ratio	0.71 (0.51, 1.17)	1.01 (0.68, 1.18)	0.68 (0.54, 0.97)	0.73 (0.66, 0.90)
E’ velocity (cm/sec)	5.95 (5.05, 6.75)	6.60 (5.41, 8.77)*	5.48 (3.85, 6.01)	5.50 (4.76, 6.35)
A’ velocity (cm/sec)	8.59 (7.28, 10.92)	9.60 (8.28, 10.11)	9.03 (8.33, 10.09)	9.29 (8.77, 10.83)
S’ velocity (cm/sec)	7.67 (5.94, 8.66)	7.60 (6.52, 8.83)	6.35 (5.99, 8.07)	8.07 (7.23, 8.82)
E/E’	9.26 (6.62, 11.50)	8.86 (6.76, 11.87)	11.11 (7.25, 14.64)	8.71 (6.62, 11.40)
RVSP (mmHg)	24.1 (21.7, 29.2)	26.0 (22.9, 27.8)	23,7 (19.2, 28.6)	24.8 (23.3, 27.8)

*P<0.05 vs. baseline values

Values are median (interquartile range).

CI, cardiac index; CO, cardiac output; EDV, end-diastolic volume; EF, ejection fraction; ESV, end-systolic volume; IVS, interventricular septal thickness; LAESVI; left atrial end-systolic volume index; LVDD, left ventricular end-diastolic dimension; LVGLS, left ventricular global longitudinal strain; LVMI, left ventricular mass index; LVPW, left ventricular posterior wall thickness; LVSD, left ventricular end-systolic dimension; RVSP, right ventricular systolic pressure; RWT, relative wall thickness; SV, stroke volume

Brachial SBP and DBP, as well as central DBP were significantly lower in the CR group. However, brachial PP, SVR, SVRI, central SBP, central PP, AIx75, TAC, and TACI showed no significant differences between the two groups. E_LV_, E_LV_I, E_A_, E_A_I, VAC, Zc, and RM also demonstrated no significant differences (S3 Table).

When compared with the baseline hemodynamic data, brachial SBP and DBP, as well as central DBP were significantly increased, while E_A_, E_A_I, E_LV_, E_LV_I, VAC, Zc, and RM were not significantly changed in the entire patient cohort (S4 Table). However, E_A_ and E_A_I were significantly decreased only in the CR group (median E_A_ decreased from 1.69 to 1.43 mmHg/mL; *P* = 0.017, and median E_A_I decreased from 0.90 to 0.79 mmHg/mL·m^2^; *P* = 0.025) (Table 6).

**Table 6:** Changes of hemodynamic data.

	With CR (N = 21)		Without CR (N = 8)	
	Baseline	Follow up	Baseline	Follow up
Brachial SBP (mmHg)	116 (104, 128)	119 (111, 128)	123 (118, 127)	123 (121, 143)
Brachial DBP (mmHg)	70 (66, 81)	70 (66, 76)	73 (65, 83)	78 (74, 85)
PP (mmHg)	40 (35, 49)	48 (40, 59)	44 (41, 60)	48 (44, 65)
SVR (dynes/sec/cm^-5^)	1730 (1403, 1911)	1499 (1351, 1769)	1552 (1403, 1726)	1718 (1563, 1855)
SVRI (dynes/sec/cm^-7^)	922 (777, 1006)	824 (740, 1035)	862 (742, 1068)	960 (850, 1161)
TAC (ml/mmHg)	1.80 (1.43, 2.13)	1.57 (1.25, 2.27)	1.83 (1.06, 2.04)	1.53 (1.36, 1.82)
TACI (ml/mmHg∙m^2^)	0.96 (0.77, 1.15)	0.90 (0.74, 1.19)	0.99 (0.62, 1.17)	0.86 (0.71, 1.01)
Central SBP (mmHg)	108 (94, 115)	110 (101, 117)	114 (108, 119)	118 (111, 134)
Central DBP (mmHg)	71 (67, 83)	71 (68, 77)	74 (66, 83)	79 (75, 86)
Central PP (mmHg)	29 (24, 42)	38 (31, 48)	36 (32, 49)	40 (35, 53)
Heart rate (/min)	65 (58, 78)	60 (54, 67)*	63 (55, 65)	59 (53, 63)
AIx75 (%)	17.8 (10.5, 25.1)	18.2 (12.9, 24.0)	17.4 (13.4, 35.4)	19.8 (10.7, 23.3)
E_LV_ (mmHg/ml)	1.62 (1.46, 1.92)	1.57 (1.25, 1.70)	1.61 (1.37, 1.71)	1.59 (1.27, 1.68)
E_LV_I (mmHg/ml∙m^2^)	0.88 (0.76, 1.04)	0.81 (0.65, 1.02)	0.89 (0.79, 0.94)	0.87 (0.70, 0.98)
E_A_ (mmHg/ml)	1.69 (1.41, 2.07)	1.43 (1.18, 1.73)*	1.54 (1.27, 2.00)	1.51 (1.42, 1.72)
E_A_I (mmHg/ml∙m^2^)	0.90 (0.76, 1.13)	0.79 (0.63, 1.03)*	0.84 (0.68, 1.17)	0.85 (0.81, 0.91)
VAC	1.05 (0.87, 1.23)	1.04 (0.84, 1.12)	0.95 (0.84, 1.17)	1.07 (0.90, 1.25)
Zc (x 10^3^ dyne-sec/cm^3^)	0.163 (0.125, 0.262)	0.248 (0.185, 0.284)	0.278 (0.150, 0.401)	0.253 (0.181, 0.309)
RM	0.83 (0.78, 0.86)	0.81 (0.77, 0.85)	0.82 (0.75, 0.88)	0.80 (0.74, 0.83)

*P<0.05 vs. baseline values

Values are median (interquartile range).

AIx75, augmentation index corrected at heart rate 75/min; DBP, diastolic blood pressure; E_A_, effective arterial elastance; E_A_I, effective arterial elastance index; E_LV_, left ventricular end-systolic elastance; E_LV_I, left ventricular end-systolic elastance index; PP, pulse pressure; RM, reflection magnitude; SBP, systolic blood pressure; SVR, systemic vascular resistance; SVRI, systemic vascular resistance index; TAC, total arterial compliance; TACI, total arterial compliance index; VAC, ventricular arterial coupling; Zc, characteristic impedance

**Fig 2:**
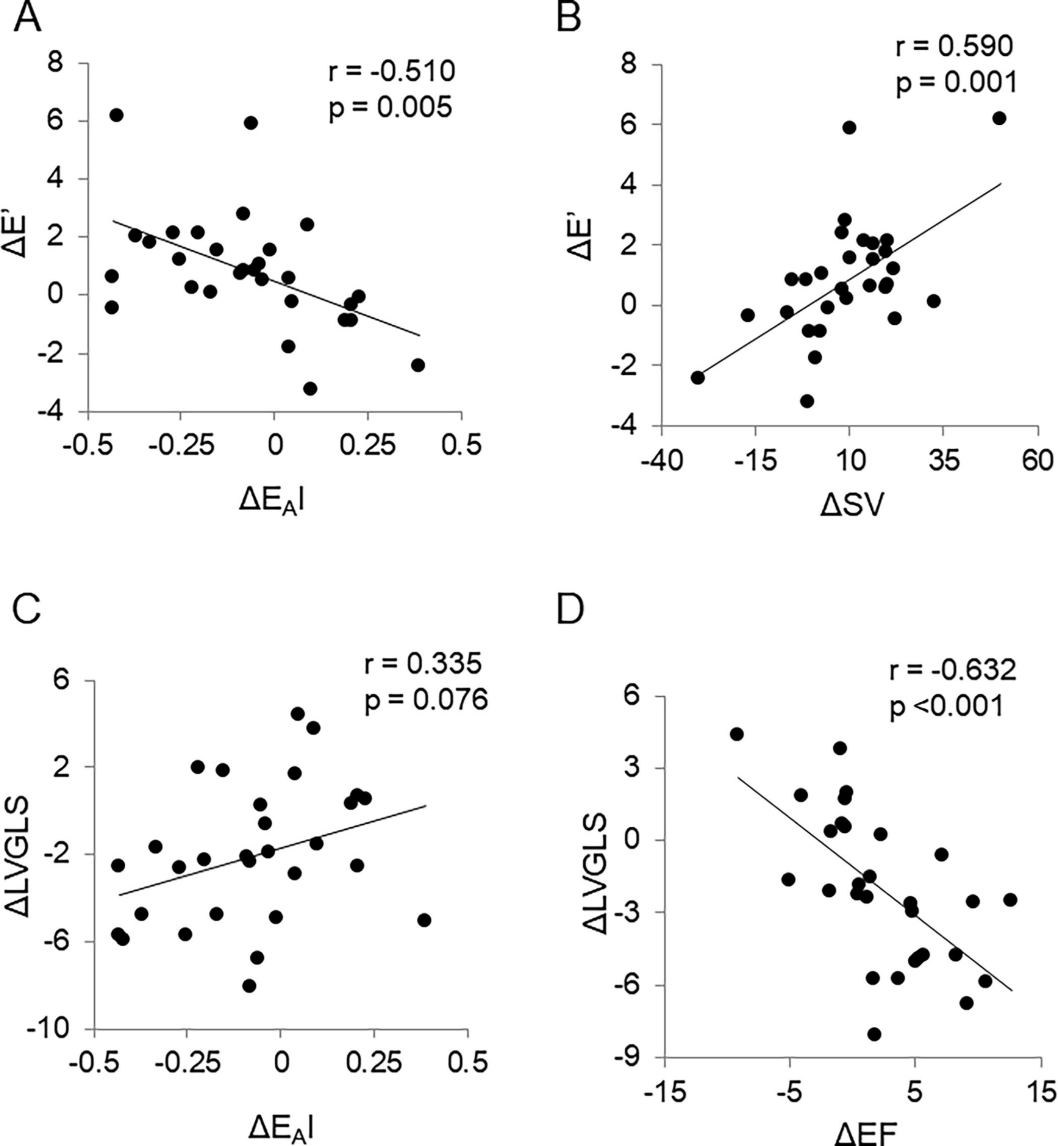
Correlations of changes in E’ velocity (ΔE’, in cm/sec)) and left ventricular global longitudinal strain (ΔLV GLS, in %) with changes in echocardiographic and hemodynamic data in the entire patient cohort (N = 29). (A) ΔE’ velocity and changes in effective arterial elastance index (ΔE_A_I, in mmHg/ml∙m^2^). (B) ΔE’ velocity and changes in stroke volume (ΔSV, in mL). (C) ΔLV GLS and ΔE_A_I. (D) ΔLV GLS and changes in ejection fraction (ΔEF, in %).

Changes in E’ (ΔE’) velocity were negatively correlated with changes in E_A_I (ΔE_A_I) and positively correlated with changes in SV (ΔSV) in the entire patient cohort (Fig 2A and 2B). In linear regression analysis, adjusted for age and sex, ΔE’ velocity demonstrated significant associations with ΔE_A_I (beta -0.424; *P* = 0.033), and ΔSV (beta 0.523; *P* = 0.004). On the other hand, ΔLV GLS was not significantly correlated with ΔE_A_I, but a negative correlation between ΔLV GLS and changes in EF (ΔEF) was found (Fig 2C and 2D) and this association remained significant in linear regression analysis, adjusted for age and sex (beta -0.605; *P* <0.004) (Table 7).

**Table 7:** Linear regression analysis of changes of E’ and LVGLS with hemodynamic data (N = 29).

	ΔE’				ΔLVGLS			
	Unadjusted		Adjusted*		Unadjusted		Adjusted*	
	Beta	*P* value	Beta	*P* value	Beta	*P* value	Beta	*P* value
ΔE_A_	-0.102	0.600	-0.248	0.207	-0.054	0.781	0.079	0.687
ΔE_A_I	-0.510	0.005	-0.424	0.033	0.335	0.076	0.185	0.363
ΔSV	0.590	0.001	0.523	0.004	-0.301	0.113	-0.192	0.313
ΔESP	0.080	0.681	0.130	0.494	0.137	0.478	0.094	0.615
ΔEF	0.286	0.132	0.259	0.157	-0.632	<0.001	-0.605	<0.001
ΔESV	0.106	0.584	0.133	0.479	0.115	0.553	0.091	0.623

*adjusted for age and sex

E_A_, effective arterial elastance; E_A_I, effective arterial elastance index; E_LV_, left ventricular end-systolic elastance; E_LV_I, left ventricular end-systolic elastance index; LVGLS, left ventricular global longitudinal strain; PWV, pulse wave velocity; SV, stroke volume; SVR, systemic vascular resistance; SVRI, systemic vascular resistance index; TAC, total arterial compliance; TACI, total arterial compliance index; VAC, ventricular arterial coupling; Zc, characteristic impedance

## Discussion

The primary findings of this study indicate that in patients with AMI and preserved or mildly reduced EF, increased aortic afterload and ventricular-arterial (VA) mismatch were associated with a negative impact on both LV diastolic and systolic function. Additionally, the outpatient CR program not only reduced aortic afterload but also improved LV diastolic and systolic dysfunction.

At first, the clinical application of VAC was limited because it required invasive catheterization and changes in cardiac preload to collect PV loops and end-systolic PV relationship for the acquisition of VAC and its components. However, it was later demonstrated that E_A_ and E_LV_ could be computed non-invasively using brachial BP, LV EF, SV and tNd [17–20], which are usually obtained during an echocardiographic study.

VAC has been reported to influence LV performance and efficiency. The evaluation of VAC helps in assessing the impact of changes in arterial properties or ventricular function on overall cardiac performance [11,21]. This is particularly relevant in the pathogenesis of heart failure. VAC has been demonstrated to be associated with the risk of hospitalization in patients with chronic systolic HF [22].

One of the non-invasive, accurate measures of myocardial diastolic relaxation is the E’ velocity of the mitral annulus [23,24]. A low E’ velocity effectively indicates LV diastolic dysfunction from its early stages. It was reported that arterial load showed an inverse association with E’ velocity [25]. In this study, E’ velocity was negatively associated with SVRI and E_A_I, indicating the impact of aortic afterload on LV diastolic dysfunction. Additionally, E’ velocity was negatively associated with VAC, suggesting that VA mismatch affects LV diastolic function.

When measuring LV systolic function, LV GLS obtained with speckle tracking echocardiography is known to be more sensitive than LVEF and capable of detecting damage to the subendocardial longitudinal fibers in patients with myocardial ischemia [26,27]. LV EF may not be a reliable prognostic index in patients with AMI and preserved EF, while it has been shown that LV GLS >-14% predicted a poor prognosis [28]. In this study, considering that median EF of patients was 53.4% (interquartile range: 49.3 ~ 59.9%) and EF was included as a component in the calculation of E_LV_, LV GLS was chosen as the LV systolic parameter to assess its association with parameters of aortic afterload. LV GLS was positively correlated with E_A_ and E_A_I, indicating the impact of aortic afterload on LV systolic dysfunction. While VA mismatch was weakly associated with impaired LV GLS, it did not reach statistical significance.

PWA is commonly used to assess arterial stiffness. The AIx, calculated by dividing the augmented pressure by aortic PP, has been reported to reflect the presence and severity of coronary artery disease (CAD) in patients under 60 years of age [29]. However, AIx is influenced by various factors beyond the amplitude of the reflected wave, such as the location of the reflected wave’s arrival, heart rate, and height. This complexity makes it challenging to determine its predictive value in clinical events [7]. Another novel index of PWA is RM, which requires the separation of arterial wave into incident and reflected waves after simultaneous recording of both arterial pressure and flow waveforms. RM has been reported as a predictor of heart failure in the general population [30,31]. Therefore, RM may serve as a more accurate index for assessing the relationship between arterial stiffness and pulsatile afterload compared to AIx. However, in this study, RM was not found to be associated with either LV diastolic or systolic dysfunction.

Impedance is calculated by dividing the modulus of pressure by the modulus of flow, obtained from the harmonic transformation of pressure and flow waveforms into a Fourier series. The input impedance (Zin) calculated from measured aortic pressure and flow waveform is not a true intrinsic impedance of the aorta because it is influenced by the reflection waves, which is determined by the overall arterial stiffness. Zc can be obtained only under conditions where peripheral arterioles are maximally dilated with minimal wave reflection, which is not always feasible. Therefore, in most studies, Zc is calculated as the average of the third to tenth harmonics of Zin [30]. However, Zc was not associated with either LV diastolic or systolic dysfunction in this study.

In this study involving patients with AMI, it was determined that E_A_I and SVRI serve as more valuable indicators of aortic afterload when compared to TACI, RM, or Zc. Notably, E_A_I faces criticism as a lumped parameter representing both aortic resistive and pulsatile load, and research has demonstrated its inadequacy in accurately reflecting pulsatile aortic load [9,32]. Collectively, these findings suggest that, within the context of AMI, the resistive or steady component of aortic load may play a pivotal role in influencing left ventricular diastolic or systolic function.

Exercise-based CR has been reported to reduce all-cause mortality, cardiovascular mortality, hospitalization, and improve the quality of life in patients with CAD, including MI [33,34]. It was demonstrated that home-based CR is non-inferior to hospital-based CR [35]. Exercise-based CR has demonstrated efficacy in improving arterial stiffness among patients with CAD [36]. Moreover, the improvement of arterial stiffness was found to correlate with changes in maximal oxygen uptake during cardiopulmonary exercise testing [37]. In HF patients with reduced EF ≤45%, CR was associated with improved VAC and mechanical efficiency [38]. CR in patients with MI was also associated with improvements in LV diastolic function, as well as LV systolic function, including EF and regional wall motion abnormality, as assessed using echocardiography [13,39]. However, impact of CR on aortic afterload and VAC in patients with AMI and preserved or mildly reduced EF has not been evaluated yet.

In this study, patients with CR showed increased SV, improved LV EF and LV GLS, but those without CR did not. The LV diastolic functional parameter, E’ velocity was also significantly increased only in patients with CR. E_LV_, E_LV_I, VAC, Zc, and RM were not changed, but E_A_ and E_A_I decreased after CR. All of these findings implicate the favorable impact of CR on aortic afterload, LV diastolic and systolic dysfunction in patients with AMI and preserved or mildly reduced EF.

## Limitations

This study has several limitations. First, although all patients received education about controlling risk factors to prevent recurrent MI during their inpatient stay, the outpatient CR program was not randomly assigned. Patients who did not participate in the outpatient CR program may have had more co-morbid conditions, but no significant differences in the baseline characteristics were found between the CR and non-CR groups. Second, more than half of the enrolled patients did not undergo follow-up echocardiographic or hemodynamic studies. As a result, the non-CR group consisted of a small number of patients, and the potential influence of spontaneous recovery of LV systolic and diastolic functions from the stunned myocardium after AMI, independent of the impact of CR, cannot be discounted. However, non-parametric statistics revealed a reduction in aortic afterload and an improvement in LV diastolic and systolic dysfunction in patients undergoing the outpatient CR program. Moreover, the negative impact of increased aortic afterload and VA mismatch on LV function at baseline, as well as the association between decreased E_A_I and increased E’ velocity in the follow-up study, were not influenced by the small number in the non-CR group, as they were analyzed in the entire patient cohort. Third, not all patients underwent assessment of cardiorespiratory fitness and peak oxygen uptake using a treadmill exercise stress test after the outpatient CR program. Consequently, the association of exercise capacity with aortic afterload, VAC, and the improvement of LV diastolic and systolic functions could not be evaluated post the outpatient CR program. Fourth, this study included a relatively small numbers of women, in whom negative impact of arterial stiffness on LV function is known to be more pronounced, despite the similar pulse wave velocity [8]. Therefore, the results of this study needed to be interpreted cautiously, especially in women.

## Conclusions

Increased aortic afterload and VA mismatch were associated with a negative impact on both LV diastolic and systolic function. The outpatient CR program decreased aortic afterload and improved LV diastolic and systolic dysfunction, supporting its beneficial role in patients with AMI and preserved or mildly reduced EF.

## Supporting information

S1 Fig Measurement of tNd, the ratio of the pre-ejection period to the total systolic period (pre-ejection period + ejection time) of ventricular systole. tNd was acquired from the pulsed-wave Doppler tracing of left ventricular outflow tract flow at the apical 5-chamber view as the ratio of the period from ECG Q wave to flow-onset to the period from ECG Q wave to end-flow.
(TIF)

S2 Fig Measurements of aortic characteristic impedance and reflection magnitude. (A) Left ventricular outflow tract flow (LVOT) acquired from pulsed-wave Doppler echocardiography at the apical 5-chamber view. (B) Digitized data of aortic pressure and LVOT flow were aligned to calculated characteristic impedance and reflection magnitude (Refer to the main text for detailed procedures).
(TIF)

S1 Table Follow up echocardiographic data. (DOCX)

S2 Table Changes of echocardiographic data in the entire patient cohort (N = 29). (DOCX)

S3 Table Follow up hemodynamic data. (DOCX)

S4 Table Changes of hemodynamic data in the entire patient cohort (N = 29). (DOCX)

S1 Dataset Minimal raw clinical data with anonymization. (XLSX)
