# Supplementary figures and images for: Impact of cardiac rehabilitation on ventricular-arterial coupling and left ventricular function in patients with acute myocardial infarction
Source: PLoS One. 2024 Apr 4;19(4):e0300578. doi: 10.1371/journal.pone.0300578 (PMC10994279; doi:10.1371/journal.pone.0300578)

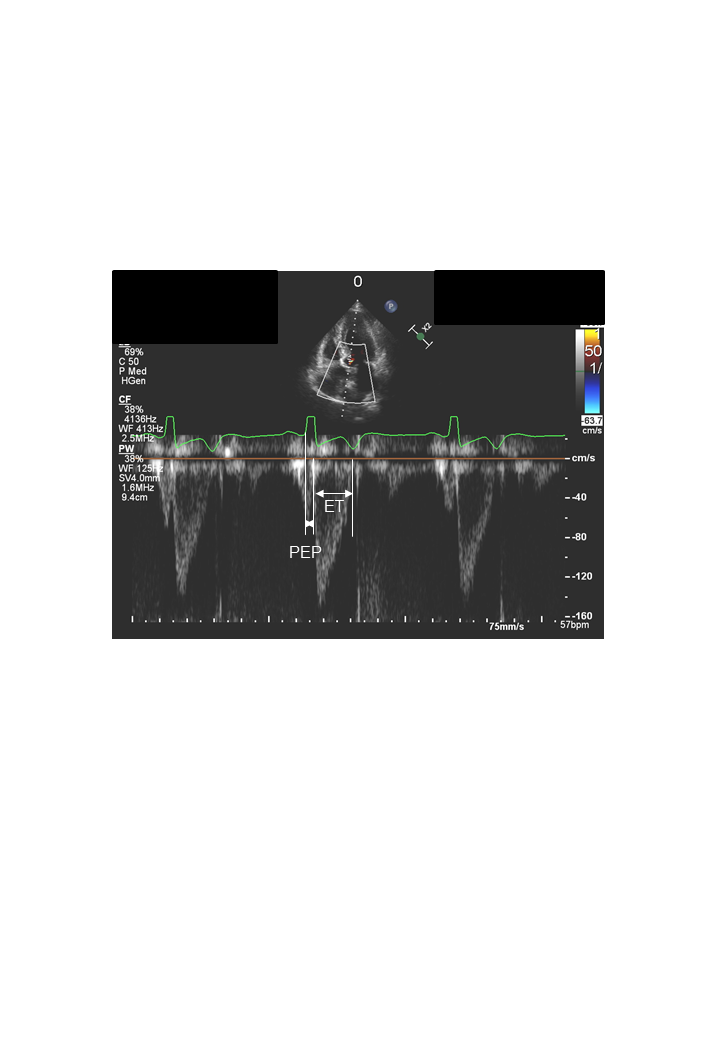

Supplement: S1 Fig — tNd was acquired from the pulsed-wave Doppler tracing of left ventricular outflow tract flow at the apical 5-chamber view as the ratio of the period from ECG Q wave to flow-onset to the period from ECG Q wave to end-flow. (TIF) [file pone.0300578.s001.tif]

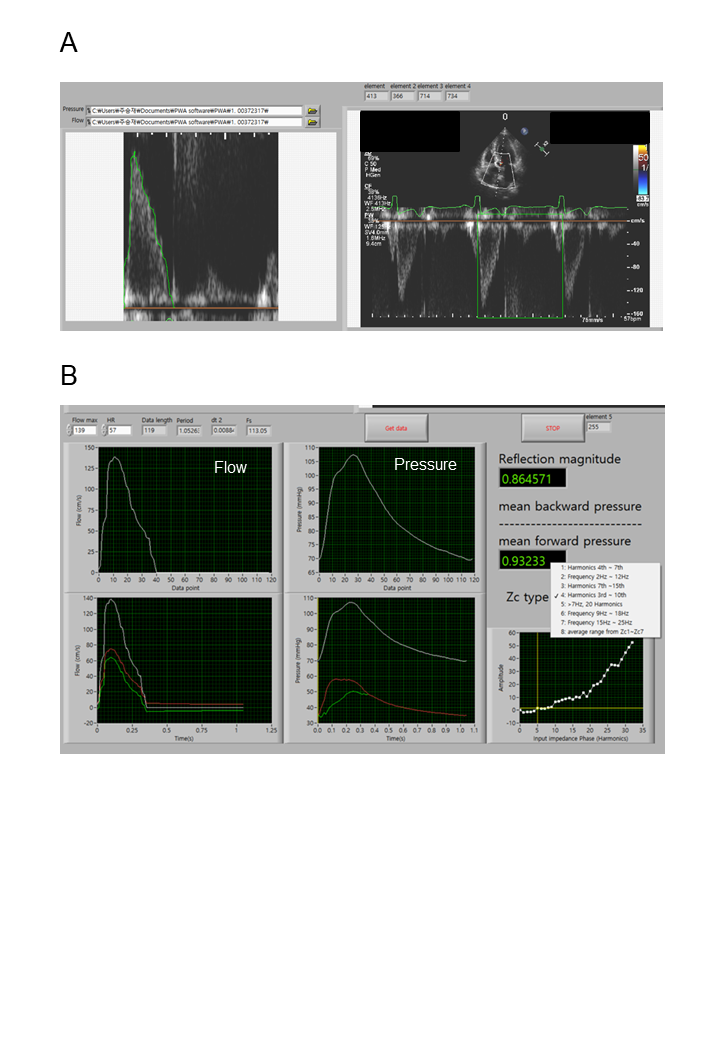

Supplement: S2 Fig — (A) Left ventricular outflow tract flow (LVOT) acquired from pulsed-wave Doppler echocardiography at the apical 5-chamber view. (B) Digitized data of aortic pressure and LVOT flow were aligned to calculated characteristic impedance and reflection magnitude (Refer to the main text for detailed procedures). (TIF) [file pone.0300578.s002.tif]
