# Supplementary material for: Impact of cardiac rehabilitation on ventricular-arterial coupling and left ventricular function in patients with acute myocardial infarction
Source: PLoS One. 2024 Apr 4;19(4):e0300578. doi: 10.1371/journal.pone.0300578 (PMC10994279; doi:10.1371/journal.pone.0300578)
Supplement: S1 Table — (DOCX) [file pone.0300578.s003.docx]

Table S1. Follow-up echocardiographic data

|  | Total (N=29) | With CR (N=21) | Without CR (N=8) | *P* value |
| --- | --- | --- | --- | --- |
| Follow up days | 272 (221, 325) | 252 (219, 325) | 283 (269, 330) | 0.429 |
| IVS (cm) | 1.00 (0.91, 1.06) | 0.98 (0.89, 1.05) | 1.03 (0.99, 1.31) | 0.184 |
| LVPW (cm) | 0.88 (0.83, 0.98) | 0.86 (0.82, 0.95) | 0.97 (0.88, 1.00) | 0.114 |
| LVDD (cm) | 4.97 (4.56, 5.20) | 4.87 (4.52, 5.23) | 5.05 (4.55, 5.17) | 0.981 |
| LVSD (cm) | 3.32 (2.90, 3.48) | 3.24 (2.90, 3.54) | 3.33 (2.93, 3.39) | 0.549 |
| LVMI (g/m^2^) | 90.2 (80.0, 102.4) | 88.4 (76.5, 97.6) | 105.2 (83.8, 124.4) | 0.103 |
| RWT | 0.37 (0.34, 0.40) | 0.36 (0.34, 0.39) | 0.39 (0.35, 0.41) | 0.257 |
| LAESVI (mL/m^2^) | 38.7 (33.5, 44.9) | 38.2 (32.0, 43.0) | 44.1 (37.5, 49.7) | 0.114 |
| EDV (mL) | 109.2 (98.4, 127.6) | 111.9 (99.8, 128.5) | 101.5 (81.1, 119.0) | 0.184 |
| ESV (mL) | 45.3 (38.4, 58.4) | 47.8 (40.3, 58.4) | 42.4 (29.6, 58.8) | 0.457 |
| EF (%) | 57.0 (52.1, 62.2) | 58.3 (52.0, 62.2) | 55.5 (52.3, 62.3) | 0.720 |
| LVGLS (%) | -15.7 (-17.6, -13.9) | -15.9 (-18.5, -14.8) | -14.0 (-16.7, -12.3) | 0.114 |
| ∆LVGLS (%) | -2.3 (-4.9, 0.4) | -2.6 (-5.4, -0.7) | -0.1 (-2.1, 3.0) | 0.011 |
| LVOTd (cm) | 2.16 (2.08, 2.32) | 2.13 (2.06, 2.22) | 2.28 (2.15, 2.37) | 0.083 |
| SV (mL) | 74.4 (67.8, 82.6) | 75.9 (65.7, 83.4) | 72.7 (70.8, 80.3) | 0.684 |
| Heart rate (/min) | 61 (56, 66) | 61 (55, 66) | 60 (55, 67) | 0.943 |
| CO (L/min) | 4.56 (4.01, 5.12) | 4.56 (3.66, 5.20) | 4.62 (4.06, 4.99) | 0.943 |
| CI (L/min/m^2^) | 2.43 (2.06, 2.88) | 2.43 (2.01, 2.86) | 2.44 (2.24, 3.12) | 0.615 |
| E velocity (cm/sec) | 60.3 (44.3, 72.9) | 63.2 (53.4, 72.9) | 45.7 (36.8, 72.3) | 0.126 |
| A velocity (cm/sec) | 71.4 (60.0, 82.1) | 71.7 (60.0, 82.8) | 68.8 (59.1, 82.0) | 0.943 |
| E/A ratio | 0.75 (0.67, 1.09) | 1.01 (0.68, 1.18) | 0.73 (0.66, 0.90) | 0.200 |
| E’ velocity (cm/sec) | 6.13 (5.32, 8.17) | 6.60 (5.41, 8.77) | 5.50 (4.76, 6.35) | 0.093 |
| ∆E’ velocity (cm/sec) | 0.69 (-0.31, 1.90) | 0.82 (-0.20, 1.90) | 0.26 (-0.34, 1.84) | 0.549 |
| A’ velocity (cm/sec) | 9.54 (8.50, 10.11) | 9.60 (8.28, 10.11) | 9.29 (8.77, 10.83) | 1.000 |
| S’ velocity (cm/sec) | 7.71 (6.81, 8.82) | 7.60 (6.52, 8.83) | 8.07 (7.23, 8.82) | 0.566 |
| E/E’ | 8.86 (6.76, 11.64) | 8.86 (6.76, 11.87) | 8.71 (6.62, 11.40) | 0.981 |
| RVSP (mmHg) | 25.2 (23.0, 27.8) | 26.0 (22.9, 27.8) | 24.8 (23.3, 27.8) | 1.000 |
| Values are median (interquartile range).  CI, cardiac index; CO, cardiac output; EDV, end-diastolic volume; EF, ejection fraction; ESV, end-systolic volume; IVS, interventricular septal thickness; LAESVI; left atrial end-systolic volume index; LVDD, left ventricular end-diastolic dimension; LVGLS, left ventricular global longitudinal strain; LVMI, left ventricular mass index; LVOTd, left ventricular outflow tract diameter; LVPW, left ventricular posterior wall thickness; LVSD, left ventricular end-systolic dimension; RVSP, right ventricular systolic pressure; RWT, relative wall thickness; SV, stroke volume | | | | |
