# Supplementary material for: Impact of cardiac rehabilitation on ventricular-arterial coupling and left ventricular function in patients with acute myocardial infarction
Source: PLoS One. 2024 Apr 4;19(4):e0300578. doi: 10.1371/journal.pone.0300578 (PMC10994279; doi:10.1371/journal.pone.0300578)
Supplement: S2 Table — (DOCX) [file pone.0300578.s004.docx]

Table S2. Changes of echocardiographic data in the entire patient cohort (N=29)

|  | Baseline | Follow up | *P* value |
| --- | --- | --- | --- |
| IVS (cm) | 1.02 (0.98, 1.11) | 1.00 (0.91, 1.06) | 0.049 |
| LVPW (cm) | 0.93 (0.86, 1.00) | 0.88 (0.83, 0.98) | 0.274 |
| LVDD (cm) | 4.90 (4.68, 5.12) | 4.97 (4.56, 5.20) | 0.430 |
| LVSD (cm) | 3.28 (2.98, 3.42) | 3.32 (2.90, 3.48) | 0.809 |
| LVMI (g/m^2^) | 89.3 (82.7, 102.1) | 90.2 (80.0, 102.4) | 0.222 |
| RWT | 0.38 (0.37, 0.40) | 0.37 (0.34, 0.40) | 0.347 |
| LAESVI (mL/m^2^) | 35.2 (31.7, 42.2) | 38.7 (33.5, 44.9) | 0.482 |
| EDV (mL) | 109.7 (85.3, 135.7) | 109.2 (98.4, 127.6) | 0.417 |
| ESV (mL) | 48.7 (40.4, 59.0) | 45.3 (38.4, 58.4) | 0.034 |
| EF (%) | 53.4 (49.3, 59.9) | 57.0 (52.1, 62.2) | 0.014 |
| LV GLS (%) | -14.4 (-15.9, -12.2) | -15.7 (-17.6, -13.9) | 0.002 |
| SV (mL) | 65.3 (55.0, 74.1) | 74.4 (67.8, 82.6) | 0.001 |
| Heart rate (/min) | 66 (57, 77) | 61 (56, 66) | 0.004 |
| CO (L/min) | 4.25 (3.66, 4.97) | 4.56 (4.01, 5.12) | 0.206 |
| CI (L/min/m^2^) | 2.28 (1.96, 2.85) | 2.43 (2.06, 2.88) | 0.139 |
| E velocity (cm/sec) | 53.6 (47.9, 66.2) | 60.3 (44.3, 72.9) | 0.157 |
| A velocity (cm/sec) | 72.9 (56.7, 84.2) | 71.4 (60.0, 82.1) | 0.430 |
| E/A ratio | 0.69 (0.51, 1.06) | 0.75 (0.67, 1.09) | <0.001 |
| E’ velocity (cm/sec) | 5.79 (5.05, 6.64) | 6.13 (5.32, 8.17) | 0.028 |
| A’ velocity (cm/sec) | 8.64 (7.66, 10.30) | 9.54 (8.50, 10.11) | 0.180 |
| S’ velocity (cm/sec) | 7.37 (5.98, 8.38) | 7.71 (6.81, 8.82) | 0.215 |
| E/E’ | 9.52 (6.67, 12.08) | 8.86 (6.76, 11.64) | 0.596 |
| RVSP (mmHg) | 24.0 (21.1, 29.2) | 25.2 (23.0, 27.8) | 0.320 |
| **P*<0.05 vs. baseline values  Values are median (interquartile range).  CI, cardiac index; CO, cardiac output; EDV, end-diastolic volume; EF, ejection fraction; ESV, end-systolic volume; IVS, interventricular septum thickness; LAESVI; left atrial end-systolic volume index; LVDD, left ventricular end-diastolic dimension; LVGLS, left ventricular global longitudinal strain; LVMI, left ventricular mass index; LVPW, left ventricular posterior wall thickness; LVSD, left ventricular end-systolic dimension; RVSP, right ventricular systolic pressure; RWT, relative wall thickness; SV, stroke volume | | | |
