# Supplementary material for: Impact of cardiac rehabilitation on ventricular-arterial coupling and left ventricular function in patients with acute myocardial infarction
Source: PLoS One. 2024 Apr 4;19(4):e0300578. doi: 10.1371/journal.pone.0300578 (PMC10994279; doi:10.1371/journal.pone.0300578)
Supplement: S3 Table — (DOCX) [file pone.0300578.s005.docx]

Table S3. Follow-up hemodynamic data

|  | Total (N=29) | With CR (N=21) | Without CR (N=8) | *P* value |
| --- | --- | --- | --- | --- |
| Brachial SBP (mmHg) | 121 (114, 132) | 119 (111, 128) | 123 (121, 143) | 0.047 |
| Brachial DBP (mmHg) | 72 (68, 80) | 70 (66, 76) | 78 (74, 85) | 0.007 |
| PP (mmHg) | 48 (42, 59) | 48 (40, 59) | 48 (44, 65) | 0.615 |
| SVR (dynes/sec/cm^-5^) | 1578 (1386, 1825) | 1499 (1351, 1769) | 1718 (1563, 1855) | 0.200 |
| SVRI (dynes/sec/cm^-7^) | 848 (749, 1071) | 824 (740, 1035) | 960 (850, 1161) | 0.168 |
| TAC (ml/mmHg) | 1.56 (1.32, 2.13) | 1.57 (1.25, 2.27) | 1.53 (1.36, 1.82) | 0.549 |
| TACI (ml/mmHg∙m^2^) | 0.88 (0.74, 1.14) | 0.90 (0.74, 1.19) | 0.86 (0.71, 1.01) | 0.582 |
| Central SBP (mmHg) | 112 (107, 120) | 110 (101, 117) | 118 (111, 134) | 0.059 |
| Central DBP (mmHg) | 73 (69, 81) | 71 (68, 77) | 79 (75, 86) | 0.010 |
| Central PP (mmHg) | 38 (32, 48) | 38 (31, 48) | 40 (35, 53) | 0.457 |
| Heart rate (/min) | 60 (54, 65) | 60 (54, 67) | 59 (53, 63) | 0.429 |
| AIx75 (%) | 19.4 (12.9, 23.7) | 18.2 (12.9, 24.0) | 19.8 (10.7, 23.3) | 1.000 |
| E_LV_ (mmHg/ml) | 1.57 (1.25, 1.67) | 1.57 (1.25, 1.70) | 1.59 (1.27, 1.68) | 0.981 |
| E_LV_I (mmHg/ml∙m^2^) | 0.82 (0.66, 0.98) | 0.81 (0.65, 1.02) | 0.87 (0.70, 0.98) | 0.649 |
| E_A_ (mmHg/ml) | 1.47 (1.28, 1.71) | 1.43 (1.18, 1.73) | 1.51 (1.42, 1.72) | 0.324 |
| E_A_I (mmHg/ml∙m^2^) | 0.83 (0.67, 0.96) | 0.79 (0.63, 1.03) | 0.85 (0.81, 0.91) | 0.324 |
| ∆ E_A_I (mmHg/ml∙m^2^) | -0.06 (-0.24, 0.07) | -0.08 (-0.26, 0.01) | 0.07 (-0.16, 0.18) | 0.153 |
| VAC | 1.04 (0.89, 1.14) | 1.04 (0.84, 1.12) | 1.07 (0.90, 1.25) | 0.324 |
| Zc (x 10^3^ dyne-sec/cm^3^) | 0.248 (0.185, 0.288) | 0.248 (0.185, 0.284) | 0.253 (0.181, 0.309) | 0.756 |
| RM | 0.81 (0.77, 0.85) | 0.81 (0.77, 0.85) | 0.80 (0.74, 0.83) | 0.401 |
| Values are median (interquartile range).  AIx75, augmentation index corrected at heart rate 75/min; DBP, diastolic blood pressure; E_A_, effective arterial elastance; E_A_I, effective arterial elastance index; E_LV_, left ventricular end-systolic elastance; E_LV_I, left ventricular end-systolic elastance index; PP, pulse pressure; RM, reflection magnitude; SBP, systolic blood pressure; SVR, systemic vascular resistance; SVRI, systemic vascular resistance index; TAC, total arterial compliance; TACI, total arterial compliance index; VAC, ventricular arterial coupling; Zc, characteristic impedance | | | | |
