# Supplementary material for: Impact of cardiac rehabilitation on ventricular-arterial coupling and left ventricular function in patients with acute myocardial infarction
Source: PLoS One. 2024 Apr 4;19(4):e0300578. doi: 10.1371/journal.pone.0300578 (PMC10994279; doi:10.1371/journal.pone.0300578)
Supplement: S4 Table — (DOCX) [file pone.0300578.s006.docx]

Table S4. Changes of hemodynamic data in the entire patient cohort (N=29)

|  | Baseline | Follow up | *P* value |
| --- | --- | --- | --- |
| Brachial SBP (mmHg) | 119 (104, 128) | 121 (114, 132) | 0.182 |
| Brachial DBP (mmHg) | 70 (66, 82) | 72 (68, 80) | 0.694 |
| PP (mmHg) | 41 (37, 51) | 48 (42, 59) | 0.090 |
| SVR (dynes/sec/cm^-5^) | 1646 (1403, 1866) | 1578 (1386, 1825) | 0.658 |
| SVRI (dynes/sec/cm^-7^) | 913 (773, 1006) | 848 (749, 1071) | 0.991 |
| TAC (ml/mmHg) | 1.82 (1.38, 2.07) | 1.56 (1.32, 2.13) | 0.770 |
| TACI (ml/mmHg∙m^2^) | 0.96 (0.73, 1.15) | 0.88 (0.74, 1.14) | 0.854 |
| Central SBP (mmHg) | 110 (96, 117) | 112 (107, 120) | 0.106 |
| Central DBP (mmHg) | 71 (66, 83) | 73 (69, 81) | 0.648 |
| Central PP (mmHg) | 33 (26, 42) | 38 (32, 48) | 0.064 |
| Heart rate (/min) | 65 (58, 78) | 60 (54, 65) | 0.001 |
| AIx75 (%) | 17.8 (13.4, 26.1) | 19.4 (12.9, 23.7) | 0.848 |
| E_LV_ (mmHg/ml) | 1.61 (1.42, 1.85) | 1.57 (1.25, 1.67) | 0.082 |
| E_LV_I (mmHg/ml∙m^2^) | 0.88 (0.78, 1.00) | 0.82 (0.66, 0.98) | 0.230 |
| E_A_ (mmHg/ml) | 1.67 (1.34, 2.06) | 1.47 (1.28, 1.71) | 0.050 |
| E_A_I (mmHg/ml∙m^2^) | 0.90 (0.75, 1.13) | 0.83 (0.67, 0.96) | 0.098 |
| VAC | 1.00 (0.87, 1.20) | -0.06 (-0.24, 0.07) | 0.721 |
| Zc (x 10^3^ dyne-sec/cm^3^) | 0.180 (0.130, 0.289) | 1.04 (0.89, 1.14) | 0.496 |
| RM | 0.83 (0.77, 0.86) | 0.248 (0.185, 0.288) | 0.289 |
| **P*<0.05 vs. baseline values  Values are median (interquartile range).  AIx75, augmentation index corrected at heart rate 75/min; DBP, diastolic blood pressure; E_A_, effective arterial elastance; E_A_I, effective arterial elastance index; E_LV_, left ventricular end-systolic elastance; E_LV_I, left ventricular end-systolic elastance index; PP, pulse pressure; RM, reflection magnitude; SBP, systolic blood pressure; SVR, systemic vascular resistance; SVRI, systemic vascular resistance index; TAC, total arterial compliance; TACI, total arterial compliance index; VAC, ventricular arterial coupling; Zc, characteristic impedance | | | |
